# Sequence variation in the human transcription factor gene *POU5F1*

**DOI:** 10.1186/1471-2156-9-15

**Published:** 2008-02-06

**Authors:** Shehnaz K Hussain, Reynaldo Sequerra, Caterina Bertucci, Noel C Hastings, Mark Rieder, Stephen M Schwartz

**Affiliations:** 1 University of California, Los Angeles, Division of Cancer Prevention and Control Research, School of Public Health and Jonsson Comprehensive Cancer Center, 650 Charles E Young Drive South, Room A2-125 CHS, Box 956900, Los Angeles, CA, 90095-6900, USA; 2 Fred Hutchinson Cancer Research Center, Program in Epidemiology, Division of Public Health Sciences, Box 358080 M4-C308, 1100 Fairview Ave N., Building M, Seattle, WA 98109-1024, USA; 3 Agencourt Bioscience Corporation, 500 Cummings Center, Suite 2450, Beverly MA, 01915, USA; 4 University of Washington, Department of Genome Sciences, Box 357730, Seattle, WA 98195, USA

## Abstract

**Background:**

POU5F1 expression is required to maintain stem cell pluripotency and for primordial germ cells to retain proliferative capability in embryonic development. Recent evidence suggests that *POU5F1 *may also be a testicular germ cell carcinoma (TGCC) oncogene, and *POU5F1 *variation may influence TGCC risk. As an important first step to a genetic association study, we sought to identify all common sequence variants in an 11.3 kb region containing *POU5F1*, and to describe the linkage disequilibrium patterns, using DNA from individuals of African-descent (AD) and European-descent (ED).

**Results:**

A higher number of polymorphisms was observed in the AD (n = 102) versus ED (n = 82) population. Among the 41 observed haplotypes, 21 (51%) and 12 (29%) were unique to the AD and ED populations, respectively, while 8 (20%) were observed in both. The number of tagging polymorphisms necessary to explain at least 80% of common variation (minor allele frequency ≥ 0.10) due to the remaining untyped polymorphisms was 17 for an AD and 10 for an ED population, providing a 4.0- and 7.0-fold gain in genotyping efficiency for characterizing nucleotide variation, respectively.

**Conclusion:**

*POU5F1 *is highly polymorphic, however a smaller subset of polymorphisms can tag the observed genetic variation with little loss of information.

## Background

Mammalian totipotent cells, including the zygote and first few cells of its cleavage, have two important characteristics, i. they are able to maintain a high rate of karyotypic normal cell division, and ii. they can differentiate into every cell and tissue type throughout the organism. POU5F1 is a member of the POU (Pit-Oct-Unc) domain transcription factor family that activates expression of target genes by binding to octameric consensus sequences [1]. POU5F1 is highly expressed in these totipotent cells but is down regulated during gastrulation and in the cells that comprise the trilaminar embryo, with the exception of undifferentiated embryonic stem cells and the primordial germ cells [2]. POU5F1 is associated with stem cell pluripotency in a somewhat quantitative manner, with less than normal levels driving cells to differentiation [3]. Methylation of regulatory sequences appears to be a principal mechanism through which POU5F1 expression is shut-down in non-germ cell embryonic tissue; primordial germ cells have mechanisms (as yet undefined) that prevent POU5F1 methylation.

There is growing evidence that POU5F1 may be important in testicular germ cell cancer (TGCC). POU5F1 is highly expressed in nearly all adult seminomas, embryonal cell carcinomas, and testicular carcinoma in situ (CIS), but not childhood TGCC or spermatocytic carcinomas, or non-germ cell testicular cancers [4,5]. Gidekel et al. found that the induction of teratomas in mice, and anchorage-independent growth of NIH3T3 cells (a characteristic of cells transformed by known oncogenes, such as RAS), were both related to POU5F1 expression in a positive dose-response fashion; [2,6] this feature is reminiscent of the quantitative relationship between expression and pluripotency maintenance [3]. Notably, the efficiency of the POU5F1 effect on NIH3T3 cells was comparable to that of the RAS oncogene in the same experimental setting. Additionally, the POU5F1 binding domain was essential for this phenotype [6] Thus, POU5F1 appears likely to be a TGCC oncogene.

The gene encoding POU5F1 (*POU5F1*) is located on chromosome 6, approximately 130 kbs telomeric of the HLA-C locus and 0.6 kb centromeric of the TCF19 locus. *POU5F1 *encodes two isoforms, isoform 1 (also known as OCT-3A) and isoform 2 (also known as OCT-3B), which share 225 of their COOH-termini amino acids, differing only in their first exon [7]. One hundred and eight polymorphisms have been reported to the National Center for Biotechnology Information (NCBI) SNP Database (as of November 13, 2006) [8] mapping to the *POU5F1 *region, however, less than half of these have been validated by multiple independent submissions or were found on more than one chromosome. The International HapMap Project, data release 21, phase II [9] has also genotyped several polymorphisms in the *POU5F1 *region in individuals from various populations, however, we do not know to what extent these data represent the complete genetic variation of the region. Given the important role of POU5F1 in stem cell pluripotency, inherited variation in *POU5F1 *conceivably could contribute to the risk of TGCC and other complex phenotypes connected to stem cell pluripotency, however, to date there have been no efforts made to systematically re-sequence the entire gene region to identify all common polymorphisms and describe their linkage disequilibrium patterns.

We conducted this study to fill in several gaps in previous knowledge concerning the genetic structure of *POU5F1*. We have re-sequenced the region surrounding *POU5F1 *and identified all common polymorphic sites in a sample of individuals of African- and European-decent, compared our findings to what has been reported previously in the NCBI SNP Database, mapped polymorphic sites to evolutionarily conserved regions, statistically inferred gene-wide haplotypes, and identified tagging polymorphisms and compared them to results based on the International HapMap Project. This information will be useful for future genetic association studies of POU5F1 and TGCC or other relevant phenoytpes.

## Results

### Polymorphism identification

**Figure 1:**
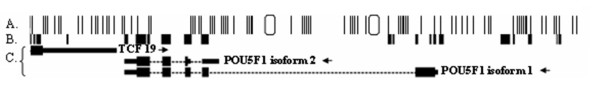
**Physical map of *POU5F1***. **A**. Lines indicate locations of polymorphisms and octagons indicate locations of two unscanned segments of intron 1. **B**. Predictions of evolutionarily conserved elements in the gene using sequences from 17 vertebrates by PhastCons (Siepel et al., 2005). **C**. Untranslated regions (thin bars), exons (thick bars), and introns (black hatched lines) of *POU5F1 *and a flanking gene, *TCF 19*. Direction of transcription is indicated by the arrows.

We identified 106 polymorphic loci in *POU5F1 *(Table 1). In the following text, these loci are named according to the number of bases from the first transcription start site, with the major allele preceding the minor allele (major allele > minor allele). Twenty-four of these loci were polymorphic in the AD population and monomorphic in the ED population, and only four loci were polymorphic in the ED population and monomorphic in the AD population. Sixty-seven percent of the AD polymorphic/ED monomorphic sites had a minor allele frequency (MAF) <0.10, whereas this was observed in only 50% of the AD monomorphic/ED polymorphic sites. Ninety-three polymorphisms (88%) were diallelic single nucleotide substitutions (SNPs) and 13 (12%) were insertion or deletion polymorphisms (indels). Most polymorphisms were located in the introns and flanking regions (Figure 1). Polymorphisms were found on an average of 1 in 103 base pairs (bp) in the AD population and 1 in 127 bp in the ED population, and a higher number of polymorphisms were observed in the AD (n = 102) versus ED (n = 82) population. No polymorphism deviated significantly (alpha < 0.05) from Hardy-Weinberg equilibrium. Seven polymorphisms were located in coding regions, however, only one was a non-synonymous SNP (4466 T > G, rs3130932). This SNP codes for a methionine to arginine amino acid substitution in the start codon of isoform 2 of POU5F1, and had a MAF of 0.23 in the AD population and 0.33 in the ED population. Within the re-sequenced region, excluding the two unscanned intronic segments, the NCBI SNP Database contained records for 108 polymorphisms. We verified 68 of these previously reported polymorphisms, failed to verify 40 polymorphisms, and identified 38 novel polymorphisms.

**Table 1:** Polymorphisms identified by *POU5F1 *re-sequencing

Site^1^	Location	NCBI rsnumber^2^	Major allele	Minor allele	Minor Allele Frequency		Site	Location	NCBI rsnumber	Major allele	Minor allele	Minor Allele Frequency	
											
					AD^3^	ED						AD	ED
-2812	5'	rs2269712	C	T	0.15	0.22	1736	Intron 1	rs3130502	C	T	0.17	0.20
-2730	5'	rs2269713	T	C	0.17	0.00	1825	Intron 1	rs9263809	T	C	0.17	0.13
-2482	5'	rs17197241	C	T	0.08	0.00	1827	Intron 1		A	G	0.02	0.07
-2411	5'	rs6910473	C	T	0.15	0.13	2524	Intron 1		C	T	0.06	0.13
-2361	5'		G	A	0.02	0.00	2578	Intron 1		T	A	0.02	0.00
-2344	5'	rs1265158	G	C	0.46	0.35	2625	Intron 1		A	C	0.06	0.13
-2218	5'	rs9501065	G	A	0.21	0.04	2665	Intron 1		+	-	0.38	0.46
-2135	5'	rs885948	C	T	0.33	0.46	2679	Intron 1		+	-	0.06	0.13
-2121	5'	rs6909939	C	T	0.15	0.13	2697	Intron 1		A	G	0.17	0.20
-1892	5'		C	T	0.02	0.00	3679	Intron 1		+	-	0.17	0.20
-1827	5'		G	C	0.04	0.00	3738	Intron 1		+	-	0.04	0.00
-1823	5'	rs885949	A	G	0.19	0.16	3781	Intron 1		T	C	0.06	0.13
-1772	5'	rs3132518	C	G	0.42	0.36	3807	Intron 1		C	T	0.17	0.13
-1755	5'	rs885950	T	G	0.40	0.48	3815	Intron 1	rs11965454	A	G	0.15	0.00
-1694	5'	rs885951	G	T	0.40	0.48	4083	Intron 1		G	A	0.06	0.13
-1691	5'	rs3132519	G	A	0.40	0.36	4202	Intron 1		C	T	0.02	0.00
-1676	5'	rs3094190	T	C	0.44	0.36	4231	Intron 1	rs3132526	A	G	0.40	0.46
-1671	5'		G	A	0.00	0.02	4284	Intron 1		T	G	0.00	0.09
-1650	5'	rs1265159	C	T	0.21	0.23	4285	Intron 1		+	-	0.08	0.17
-1611	5'	rs3132520	G	A	0.40	0.55	4322	Intron 1	rs1265163	G	C	0.17	0.20
-1566	5'	rs28383834	-^4^	+	0.04	0.09	4466	Exon 1^6^	rs3130932	T	G	0.23	0.33
-1107	5'	rs1108746	G	T	0.11	0.10	4515	Exon 1^6^	rs9501063	C	G	0.15	0.00
-1093	5'	rs885952	G	A	0.43	0.55	4539	Exon 1^6^		C	T	0.06	0.13
-1055	5'	rs879882	G	A	0.50	0.21	4746	Intron 2		A	G	0.06	0.13
-985	5'	rs5875289	-	+	0.17	0.17	4787	Intron 2	rs9281233	+	-	0.23	0.33
-734	5'	rs17197220	A	G	0.04	0.04	4834	Intron 2		+	-	0.35	0.33
-643	5'	rs9263818	G	T	0.17	0.14	4908	Intron 2	rs2106074	T	C	0.23	0.33
-520	5'	rs9263817	C	T	0.17	0.14	5724	Intron 4	rs3064866	+	-	0.23	0.33
-513	5'	rs3094191	A	G	0.10	0.23	5763	Intron 4	rs9263795	A	G	0.00	0.13
-400	5'	rs9263816	C	A	0.17	0.14	5770	Intron 4	rs2394882	G	T	0.23	0.33
-310	5'	rs17190839	C	T	0.15	0.00	6005	Exon 5	rs1061118, rs17197178	T	C	0.25	0.25
-75	5'	rs17197199	G	A	0.15	0.00	6116	3' UTR	rs3734864	G	A	0.02	0.00
21	Exon 1^5^	rs2077010	G	A	0.42	0.50	6279	3'	rs13409	C	T	0.41	0.48
27	Exon 1^5^	rs1265160	C	T	0.15	0.20	6334	3'	rs3130933	G	A	0.00	0.11
291	Exon 1	rs1062630	C	T	0.20	0.20	6466	3'		T	C	0.02	0.00
561	Intron 1	rs3130504	A	T	0.17	0.20	6582	3'	rs17190811	T	A	0.06	0.13
594	Intron 1	rs3094192	C	G	0.17	0.20	6623	3'		C	T	0.02	0.00
600	Intron 1		G	A	0.25	0.17	6712	3'	rs9501062	A	G	0.12	0.00
601	Intron 1		C	T	0.04	0.00	6808	3'		G	T	0.17	0.20
624	Intron 1	rs3094193	A	C	0.33	0.33	6810	3'		A	T	0.17	0.20
639	Intron 1	rs3132522	G	A	0.17	0.20	6813	3'		+	-	0.17	0.20
653	Intron 1	rs9263811	G	A	0.17	0.13	6822	3'		G	A	0.02	0.00
663	Intron 1		C	G	0.19	0.04	6851	3'	rs3132528	A	G	0.17	0.20
679	Intron 1		-	+	0.42	0.50	7144	3'	rs17190798	T	A	0.06	0.14
683	Intron 1		T	C	0.04	0.07	7211	3'	rs17190783	T	C	0.06	0.14
690	Intron 1		C	T	0.19	0.04	7555	3'	rs17190776	C	T	0.06	0.13
709	Intron 1		T	G	0.02	0.00	7599	3'	rs1044870	G	A	0.17	0.13
742	Intron 1		C	T	0.10	0.00	7660	3'	rs1841	T	C	0.04	0.07
770	Intron 1	rs9263810	C	G	0.17	0.13	7827	3'	rs1419881	T	C	0.44	0.50
1424	Intron 1		A	G	0.02	0.00	7845	3'	rs9501503	G	C	0.02	0.00
1570	Intron 1	rs3132523	G	A	0.17	0.20	7918	Exon^7^	rs1065461	G	A	0.17	0.20
1650	Intron 1	rs17840029	T	C	0.04	0.04	8128	Intron^7^		G	A	0.02	0.00
1688	Intron 1	rs3132524	G	A	0.17	0.20	8169	Intron^7^	rs3216869	+	-	0.12	0.00

^1^Polymorphism sites are named according to the number of bases from the first transcription start site, ^2^National Center for Biotechnology Information (NCBI) rsnumbers are included if they have been previously published, ^3^AD, African-descent and ED, European-descent populations, ^4 ^(-), deletion and (+), insertion polymorphisms. The (+) allele for each loci is as follows: -1566 = G, -985 = G, 679 = TCTG, 2665 = G, 2679 = AG, 3679 = GAG, 3738 = TG, 4284 = CTC, 4787 = CT, 4834 = GGGCTT, 5724 = TC, 6813 = A, 8169 = GAAG, ^5^Exon for POU5F1 isoform 1 only, ^6^Exon for POU5F1 isoform 2 only, ^7^Loci are 3' of the *POU5F1 *gene, and in the exon/intron of the flanking TCF 19 gene.

### Comparative genomics

There were 20 highly conserved elements among vertebrates identified in the re-sequenced region, with log-odds scores ranging from 211 to 555 (Figure 1B.). Nine polymorphisms were located in conserved regions in the exons (67%) or 5' gene regions (33%): -2482 C > T (rs17197241), -1611 G > A (rs3132520), -75 G > A (rs17197199), 21 G > A (rs2077010), 27 C > T (rs1265160), 291 C > T(rs1062630), 5763 A > G (rs9263795), 5770 G > T (rs2394882), and 6005 T > C (rs1061118, rs17197178).

### Linkage structure

Both populations displayed high haplotype diversity. In total, there were 41 haplotypes inferred among the 94 chromosomes, or 47 individuals, represented. Twenty-one (51%) of the haplotypes were unique to the AD population, 12 (29%) were unique to the ED population, and 8 (20%) of the haplotypes were shared across both populations (Table 2). A majority of the haplotypes carried exclusively in either the AD or ED populations were only observed once, 76% and 50%, respectively; the majority (75%) of haplotypes carried in both populations had a frequency ≥ 0.05. Three of the most common haplotypes accounted for 28% of all haplotypes. The non-synonymous SNP in the start codon of POU5F1 isoform 2 was carried in 18 haplotypes. Analysis of the recombination rates revealed a potential recombination hotspot in the ED population in intron 1 between loci -1755 T > G and -1694 G > T. Recombination between these two loci exceeded the background rate of recombination by a factor of 11 (data not shown).

**Table 2:** Distribution of *POU5F1 *haplotypes by population and haplotyope frequency

Population^1^	Haplotype frequency, N(%)			Total N
		
	0.02	0.04	≥ 0.05	
AD, only	16(76)	1 (5)	4 (19)	21
ED, only	6 (50)	4 (33)	2 (17)	12
AD and ED	0	2 (25)	6 (75)	8

^1^AD, African-descent, ED, European-decent populations

**Figure 2:**
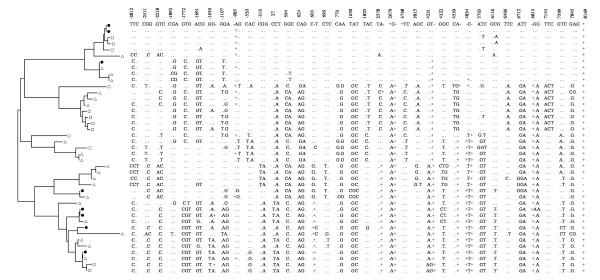
**Phylogenic tree of the 41 observed *POU5F1 *haplotypes**. The tree was created using the neighbor-joining method in MEGA (version 3.1). The branch line length is proportional to the number of nucleotide differences between haplotypes from the root. The polymorphisms in each haplotype are ordered from 5' to 3', in the same order as the polymorphisms listed in Table 2, and the first site in each triplet is listed as the column heading. The (.) indicates that the allele is the same as the first haplotype in the tree. The (-) indicates a deletion, and the (+) indicated an insertion. Haplotypes unique to the African-descent population are indicated by the white triangles, those unique to the European-descent population are indicated by white squares, and those observed in both populations are indicated by black circles.

The phylogenetic analysis of *POU5F1 *haplotypes shows that the earliest division of the haplotype tree into two large clades differentiate between one clade that mainly consists of haplotypes from AD individuals, and another that contains a mix of haplotypes from AD and ED individuals (Figure 2). Although several clades have mixed AD and ED haplotypes, often the nearest neighboring haplotypes are from the same population.

**Figure 3:**
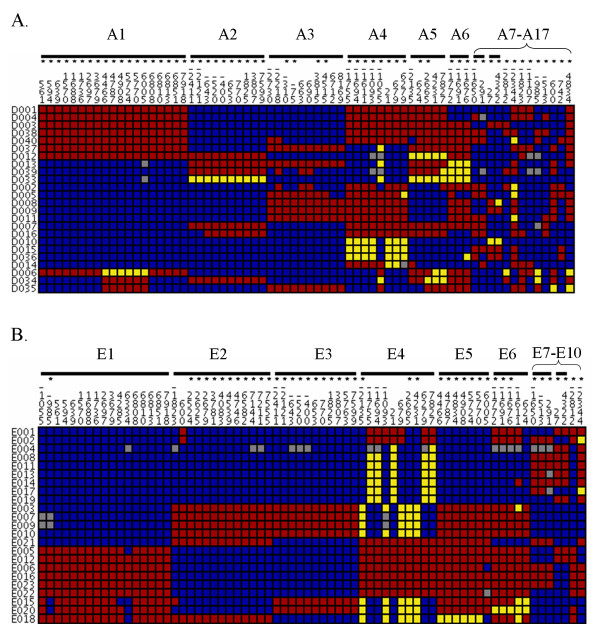
**Visual genotypes for *POU5F1 *polymorphisms in African-descent (A.) and European-descent (B.) populations**. Subjects (rows) and polymorphisms (columns, details for each polymorphism appear in Table 2) are clustered by groups of correlated polymorphisms (r^2 ^≥ 0.80), indicated by black lines. Colored squares represent genotypes at each polymorphic site: red = heterozygotes, yellow = homozygotes (minor allele), blue = homozygotes (major allele), and grey = missing data. Asterisks denote best choices for tagging polymorphism(s) based on r^2 ^values with other polymorphisms within the cluster. Only polymorphisms with a minor allele frequency ≥ 0.10 are included.

In the AD population there were 68 polymorphisms with a MAF ≥ 0.10. With an r^2 ^threshold of 0.80, these polymorphisms can be grouped into 17 clusters (9 of which are singles) which can be tagged by an equal number of tagging polymorphisms (Figure 3). In the ED population, among the 70 polymorphisms with a MAF ≥ 0.10, 10 tagging polymorphisms can be selected to capture the clusters of correlated polymorphisms, of which 2 are singletons. For a combined population of AD and ED individuals, 24 tagging polymorphisms can tag the clusters of correlated loci, due to overlap in the correlation patterns between the AD and ED populations.

The International HapMap Project genotyped 36 POU5F1 loci that were polymorphic in samples from either the population of European-decent (CEU) or Yoruban-decent from Nigeria (YRI), or both. With an r^2 ^threshold of 0.80, HapMap POU5F1 polymorphisms with a MAF ≥ 0.10 can be clustered into 9 groups in the CEU population and 12 groups in the YRI population.

## Discussion

POU5F1 has a critical role in regulating pluripotency in embryonic development [1-3,10-12]. In non-diseased tissue and tissue from non-germ cell tumors, POU5F1 expression is virtually undetectable [4,5,7,13]. However, the observation that POU5F1 is expressed in TGCC [14-16], and may be somatically mutated in other cancers [17], suggests that genetic variation in *POU5F1 *could conceivably alter protein expression or activity, thereby influencing risk of TGCC. In this paper, we have identified the presence of 38 new polymorphisms, and verified the presence of 68 previously reported polymorphisms, in the African-descent and European-decent populations in the gene region encompassing *POU5F1*. A majority of the 40 polymorphisms contained in the NCBI SNP database that we did not detect were those associated with a low level of internal validation (i.e. single submission or mutation on a single chromosome), and thus most likely represent genotyping errors or somatic mutations. Our observation that the AD population was more polymorphic compared to the ED population is consistent with prior reports [18], however the observed SNP density (1 in 103 bp in the AD population and 1 in 127 bp in the ED population) of *POU5F1 *is higher than most genes [19-21].

We analyzed linkage disequilibrium patterns in *POU5F1*, and identified clusters of correlated polymorphisms. The NCBI SNP Database contains records for at least one possible tagging polymorphism for all but three of the 17 AD clusters (A15–A17, Figure 3), and for all 10 ED clusters. The HapMap Project genotyped approximately 1/3 the number of polymorphisms as we identified in our re-sequencing of the *POU5F1 *region in populations with similar ancestry. They also observed a lower number of unique clusters of correlate polymorphisms, which suggests a less-than-complete coverage of *POU5F1 *LD patterns by the HapMap Project. Furthermore, our AD population is an admixed population and likely exhibits a more complex pattern of LD compared to the YRI HapMap population.

Within groups of correlated polymorphisms, selecting tagging polymorphisms should not only consider the level of correlation with other polymorphisms, but also the extent to which a particular SNP may have functional consequences. Several polymorphisms identified in *POU5F1 *make them potentially interesting candidate polymorphisms. For example, we confirmed the existence of a common SNP at position 4466 T > G (rs3130932) that produces a non-synonymous change in the initiating methionine (ATG → AGG) of POU5F1 isoform 2 [7]. Carriers of this variant cannot express POU5F1 isoform 2. Alternative mRNA splicing is a process that is signaled by the recognition of specific nucleotide sequences on either the proximal or distal end of the splice junction, and results in structural and functional diversity in proteins. Few data exist on expression of the different POU5F1 isoforms in germ cells or germ cell tumor tissue, since most expression studies to date have not discriminated between the two isoforms. In human preimplantation embryos, however, the two isoforms displayed temporal and spatial differences in expression [22]. While isoform 1 is expressed in the nucleus of compacted embryos and blastocysts, isoform 2 expression was only weakly detected in the periphery, or cytoplasm, of zygotes suggesting that the isoform 2 may have other functions beyond transcription activation [22].

Other potentially interesting regions of the gene are evolutionarily conserved elements. POU5F1 expression is thought to be driven by a promoter lacking a typical TATA box, and having at least one distal and one proximal enhancer that are maintained in conserved regions [23]. Within this promoter region, there are four regions of homology between species with 66% to 94% conservation of bases [24]. We observed 3 SNPs mapping to these conserved regions, -2482 C > T, -1892 C > T, and -75 G > A with minor allele frequencies of 0.08, 0.02, and 0.15 in the AD population and no nucleotide variation in the ED population. If the presence of these variant alleles disrupts binding of regulatory elements, and therefore reduces POU5F1 expression, these variants may help to explain part of the lower risk of TGCC observed in the AD population [25].

One limitation of our study is that our population was too small to identify rare polymorphisms reliably. However, by studying 46 ED chromosomes and 48 AD chromosomes, we have 90% power to detect polymorphisms with a minor allele frequency of at least 0.05 in each population [26]. Thus, we have excellent power to detect common variation, which is generally the most useful for typical genetic association studies that involve hundreds, as opposed to thousands, of cases. However, we did not scan two intronic segments, totaling 1 kilobase pairs (kbs), for genetic variants. NCBI contains records for 24 polymorphisms mapping to these intronic regions, so it is possible that we have missed some common variants that existed in our study population in these regions, and also that these variants tag unique patterns of variation that we did not identify. A second limitation is that we only studied two populations, and our observed variation and linkage disequilibrium patterns may not extend to individuals in other populations, for example Asians. The HapMap Project, which includes Japanese, Chinese, African, and CEPH samples, shows that allele frequencies and linkage disequilibrium patterns for POU5F1 polymorphisms vary between populations, indicating that future resequencing projects in other population may be needed.

Further studies are also needed to evaluate the functional relevance of these polymorphisms, as well as investigations into whether these polymorphisms impact TGCC risk or other phenotypes related to stem cell pluripotency.

## Conclusion

This paper provides the first comprehensive report of sequence variation in an 11.3 kbs region containing *POU5F1*. We identified 106 polymorphisms, 36% of which have not previously been reported. Several polymorphisms were identified in conserved regions, including a non-synonymous polymorphism occurring in one-quarter and one-third of the AD and ED populations, respectively, that causes a methionine to arginine amino acid substitution in an alternative start codon of POU5F1. Linkage disequilibrium patterns differed by population, however, a much smaller subset of polymorphisms may be used to tag the observed common genetic variation in either population with little loss of information, amounting to a 4.0- and 7.0-fold gain in efficiency in the AD and ED populations, respectively.

## Methods

### Samples

Twenty-four samples from unrelated individuals of African-descent (AD) and 23 Centre d'Etude de Polymorphism Humain (CEPH) samples from unrelated individuals of European-descent (ED), were obtained from the Coriell Repository (Camden, New Jersey, USA) and used for gene re-sequencing (sample identification numbers for the AD population: NA17101, NA17102, NA17103, NA17104, NA17105, NA17106, NA17107, NA17108, NA17109, NA17110, NA17111, NA17112, NA17113, NA17114, NA17115, NA17116, NA17133, NA17134, NA17135, NA17136, NA17137, NA17138, NA17139, NA17140; for the ED population: NA12560, NA12547, NA10845, NA10853, NA10860, NA10830, NA10842, NA10851, NA07349, NA10857, NA10858, NA10848, NA12548, NA10844, NA10854, NA10861, NA10831, NA10843, NA10850, NA07348, NA10852, NA06990, NA07019).

### Gene re-sequencing

Approximately 11.3 kpb of DNA containing the POU5F1 gene (3.0 kbp 5' of the gene, 6.0 kbp of the gene, and 2.3 kbp 3' of the gene) were amplified and scanned for variation using high-throughput methods. Primers were designed for 25 amplicons of approximately 600 bp each covering all exons, flanking regions, and most intronic regions (Table 3). To ensure that POU5F1 was targeted specifically, and not a pseudogene, extra care was taken to design primers and amplicons using Agencourt's proprietary modeling software. During the modeling stage, an *in silico *ePCR check was done on each amplicon to verify that each amplicon only had one ePCR hit. A positive ePCR hit is defined as having the forward and reverse primers match the genomic sequence exactly without a single mismatch. Two segments of the first intron totaling approximately 1 kb were not scanned. Universal forward (GTAAAACGACGGCCAGT) or reverse (CAGGAAACAGCTATGACC) sequences were added to the 5' end of primers to generate fragments compatible with dye-primer florescence-based sequencing. Overlapping primers were designed to identify any instances of primer specific amplification. PCR products were purified utilizing Agencourt's solid phase reversible immobilization reagent, AMPure. Bi-directional (forward and reverse) sequencing of PCR products was performed using the ABI BigDye Terminator v3.1 cycle sequencing chemistry, followed by purification using Agencourt CleanSeq reagent. Determination of sequencing products was performed using ABI 3700/3730 capillary sequencers. Sequencing reactions were performed twice in each direction, resulting in four traces for each amplicon.

**Table 3:** Nucleotide sequences for the forward (F) and reverse (R) primers used in the *POU5F1 *sequencing reactions

Oligo Name	Sequence	Oligo Name	Sequence
01F	GCTGCTCATCTCACCTCTCC	14R	AGGTGATTGTTTGAGCAAAGG
01R	AAACACCTTCCCCAATTTCC	15F	AGATGGAGTCTCACTCTGTC
02F	GCTCATATCCAGCCACAAGG	15R	TTGAGCCCAAGAGTTAGAAAGC
02R	AATACCTGCCACAGGTCTGC	16F	GGCCTCCCAAAGTGAAGG
03F	ACCAGGCCCCATAATCTACC	16R	TGCATACACACAAACACAGC
03R	TCATGCTGCTGGTCTAGTGC	17F	GATGTCAGGGCTCTTTGTCC
04F	GAGCCCCCAGACTTACCC	17R	AAAGAAGATAGTTCATTTAATACCTGCAAAA
04R	AGAGGGGGCAGCTCTAACC	18F	GATCTCAGCTCACTGCAACC
05F	CTCCTCTGCGTCTTTCTGC	18R	TCGGGATTCAAGAACCTACG
05R	CTGCACATCAGGTTCCTTGC	19F	TGGGTGAATGACATTTGTGG
06F	CTCCCAGGCTTCTTTGAACC	19R	CGTTTGGCTGAATACCTTCC
06R	AATCCTAGGCATTCCCATCC	20F	TCTGGGAAGAGGTGGTAAGC
07F	GTGCTTATGGCTGTTGATGC	20R	CTGGTTCGCTTTCTCTTTCG
07R	GTCTGTGGAAGGGGAAAACC	21F	AAAGCTTGCCCTTGTCACC
08F	GAGCAGAAGGATTGCTTTGG	21R	CCTCTCGTTGTGCATAGTCG
08R	GGCCTTGGAAGCTTAGCC	22F	GAATGTCCAAGCAGAGTCAGG
09F	TGAGTAGTCCCTTCGCAAGC	22R	GATGTGGGATTAAAATCAAGAGC
09R	ACCCTGCCTGCTCCTCTCC	23F	AGGAATTGGGAACACAAAGG
10F	AGGAGGCAAGTGAGCTTCG	23R	TCTGGTTGGGGTGATCTAGG
10R	GGTAAACCCAGCTCACAACG	24F	GTTCAGAGCTGGCTTTTTGC
11F	GGGTAAAAACAGTGCTCATTCC	24R	GAAGATTTATTTACAGGCTTCACC
11R	CCCCCTAGGAGATTTTGTGC	25F	GCCATCACACTGGTAAACTGC
12F	ATAATGGCTGGCAATTGTGG	25R	GCACCTATCAGTTGCAAAAGC
12R	CTGAGGATGACTGGGTTTGG	26F	GGTCGCTACGATTTTGTTGG
13F	CACCCTCAAGGCTTAAATGC	26R	CCTGGGTTCAGTGTGATGG
13R	TGAGAGTTTCGCTTTTGTTGC	27F	CGCTGCTGTGTCTACTCATGG
14F	AAAAAAGGTGAGGCTAGGTGC	27R	AACCGGAGTGAGCATGTCC

### Analysis

#### Polymorphism identification

Chromatograms of fluorescence-based sequences were compared across all samples to identify polymorphic loci and to determine which individuals were heterozygous or homozygous for the common or variant alleles using PolyPhred [27]. PolyPhred uses the base calls and peak information provided by Phred [28,29] and the sequence alignments provided by Phrap. PolyPhred detects a heterozygous locus by a significant drop in the height of a fluorescence peak along with the presence of a second fluorescence peak, but ignores regions of poor sequence quality, such as the ends of amplicons, to reduce the number of miscalled sites. In addition to using PolyPhred, all sequences were visually inspected in Consed [30] by a data analyst for confirmation. The polymorphisms were named according to distance in bases from the first translational start, according to our reference sequence [See Additional file 1], 3,055 bases downstream of our re-sequencing start site.

We accessed the National Center for Biotechnology Information (NCBI) SNP Database on November 13, 2006 [8], and compared the polymorphisms identified from our re-sequencing to those contained in the database.

#### Comparative genomics

We analyzed the polymorphism data using the University of California Santa Cruz (UCSC) Genome Browser human assembly from March 2006 [31] to identify the location of SNPs in relation to conserved elements within and flanking *POU5F1*. We used the 'most conserved' track setting which shows predictions of conserved regions using sequences from 17 vertebrates produced by PhastCons software [32]. PhastCons uses a phylogenetic hidden Markov model and a maximum-likelihood approach to produce conservation scores for each nucleotide of the alignment, which can be interpreted as the probability that the nucleotide is in a conserved element. Each predicted conserved element was assigned a log-odds score indicating how much more likely it is under the conserved phylogenetic model than the nonconserved model.

#### Linkage structure

Haplotypes were inferred separately for the AD and ED populations using PHASE (version 2.1) software, which uses a Bayesian statistical method [33]. Posterior recombination parameters, obtained from the general model for varying recombination rate in PHASE, were used to determine the presence and location of any potential recombination hotspots defined by a substantial difference in recombination between two neighboring loci and the background recombination rate [34]. Ancestral relationships between inferred haplotypes from both populations were examined using MEGA (version 3.1) software [35], using the neighbor-joining method [36]. The square of Pearson's correlation coefficient (r^2^) for pairwise comparisons of biallelic polymorphisms was used to determine the extent to which polymorphic loci provide redundant genotype information. Groups of correlated polymorphisms were identified through a binning algorithm using a threshold value of r^2 ^≥ 0.80 between one polymorphism and a maximum number of other polymorphisms, separately for the AD and ED populations, using the LDSelect version 1.0 program [37]. LDSelect also identifies desirable tagging polymorphisms for each group of polymorphisms based on the r^2 ^statistic between polymorphisms within a group. In addition, the combined population of AD and ED individuals was analyzed using the multiPopTagSelect version 1.1 program, which identifies the smallest number of maximally informative tagging polymorphisms to capture correlation patterns in both populations [38].

Within the region we resequenced, the International HapMap Project, data release 21, phase II, [9] has genotyped 31 polymorphisms in several populations (including 90 Caucasian individuals from Utah, USA, in the CEPH collection). We  compared the tagging polymorphisms identified from our genotype data to that  of two populations in the HapMap project.

## Authors' contributions

SKH participated in the coordination of the project, conducted data analysis, and drafted the manuscript. RS conducted the sequencing reactions. CB, NCH and MR analyzed the sequence data for polymorphism discovery. SMS conceived the study, obtained funding, and participated in the design and coordination. All authors read and approved the final manuscript.

## Supplementary Material

Additional file 1 Consensus Sequence For POU5F1. The data provided represent the consensus nucleotide sequence for the gene encoding POU5F1. Click here for file
