# Supplementary material for: Sequence variation in the human transcription factor gene POU5F1
Source: BMC Genet. 2008 Feb 6;9:15. doi: 10.1186/1471-2156-9-15 (PMC2275747; doi:10.1186/1471-2156-9-15)
Supplement: Additional file 1 — Consensus Sequence For POU5F1. The data provided represent the consensus nucleotide sequence for the gene encoding POU5F1. [file 1471-2156-9-15-S1.doc]

**Supplementary Table: Reference sequence**

| CTGGCTTCCA GTCTCCTCCA GGCCCAACGG TGGCCCCCAG CACTGGTTGG | -3006 |
| --- | --- |
| GGCCTGGGAG AGCTGGCCTT GGCTGAAGTG AAGCCACCTA CCCTTCAGGC | -2956 |
| ATAACAGGAC AGTGAGAAGG AAGGAAAGCC TGCCTCAACC TCCCATCAGC | -2906 |
| CCTGAGCACC CCAGAAGGGG GCCGGCTAGG AGTCTAGGCA TGCAGGAGGC | -2856 |
| TGACCCCTGA CTGGGCTCAT ATCCAGCCAC AAGGCAGCCA GGGACCCAGG | -2806 |
| CACCCACCCC TTGTCTGCGT CCCTCTCGGG AATGGGCCTC TTGCCCAGGC | -2756 |
| CAGAAATACA CCACCTACAG TACAAATTAT AATCTAAAAA CAAGAGGGTG | -2706 |
| GTGTTGAGTG GGGAAATTGG GGAAGGTGTT TTAGGAGCCA CTAGGAAAAT | -2656 |
| GGGCAGCAGG GACTCTCTGG ACTGGCTTGG GAAGAGCGCT TTTGGGGAAC | -2606 |
| CTGGAGGATG GCAAGCTGAG AAACACTGGT GTGGAGATTC CAGCCAAATC | -2556 |
| CCAGGCCTGC CCCTCCCCCT CCTCTGAGAG GCCGTCTTCT TGGCAGACAG | -2506 |
| CAGAGAGATG CATGACAAAG GTGCCGTGAT GGTTCTGTCC TGGGGATTGA | -2456 |
| GATGGCTGGG GAGGGGCCTC CTCCTGTTCC GAAGCATGTT CCTCCCACCC | -2406 |
| CCACCAGGCC CCATAATCTA CCTGCCTTTT GGGCAGTTAA AGGCCGAGAA | -2356 |
| GTGAACACAG CTGCAACCCC ACTGCCTTGT AGACCTTCCG GCAGACCTGT | -2306 |
| GGCAGGTATT GAAATGCACG CATACAATTA GGCTCAAAAA GTCTACACAG | -2256 |
| ACAGGAGATG GGCACACGAA CAGAGGCAAC ATAAGAGTGG GGGAAAAGTC | -2206 |
| TCAAAAGACT CACGGATGCC ACCAAGATGA AGACAGCTGG CCACGGGACA | -2156 |
| CCCATCCCCT TAGAAGGCAG ACAGAGCCAC TGACCCCAGC AGACAAGCCC | -2106 |
| AGGCAGGGCT GAGCCTGGAG CCTGCAATGA GAAGCCTTAC TTAAGTCGAC | -2056 |
| AGAGGTCAGC GTGCCCAGTC CAGACCTGGC CTTCTGGCCT TCGAAGCTGT | -2006 |
| GGGGAGCCCT GGCCCAGAGC CCCCTCTGGA GCCCCCAGAC TTACCCCAGG | -1956 |
| CCCTCCACTG AGATCAAGTT TTGGGAGCAG ACAGACAAAC ATCATCCCTC | -1906 |
| ACAGACAGGC ATTCCGTTGG CTATTCTCTT GCAAACAGAA TCAAGCACTA | -1856 |
| GACCAGCAGC ATGAGCCTCA GGATACTCAG GCCAGGCCCA GAAAAACAGA | -1806 |
| CCCTGAAGGG GAGCTTAGGG CAGCCTTCCT GCACCCCTCC ACAAATCACT | -1756 |
| CTCCACCTCC TCTGCGTCTT TCTGCCAGCC AGCCCCACTA AACAAAGCAC | -1706 |
| ATCCCTCAAT CTGCCGGGCT CGGGGAGGGA CGCACGATGA AGCTGGACGC | -1656 |
| CTGAGTCCCC CAGAGGAAGG AGGAACTAGA TACCTAGGTC CCTGTGGGGG | -1606 |
| GCCCTTGGTG CCCGTCTGAG GCTCAGTCTT TGAGGGGATT GCAGAGGGGG | -1556 |
| GTTGCTGGAG CTCCTTTTAG CGTCTCTGAA GGGGATTCTG TGTGAGGGGA | -1506 |
| TTGGGACTGG GGGGTTGGGG AGCAGGAAGC AGTCCCCAGG GGAGCCATCC | -1456 |
| AGGCCCATTC AAGGGTTGAG CACTTGTTTA GGGTTAGAGC TGCCCCCTCT | -1406 |
| GGGGACCGGG ATTGTCCAGC CAAGGCCATT GTCCTGCCCC CTTCCCCCAG | -1356 |
| TCCCTCCCAG GCTTCTTTGA ACCTGAAGTC AGATATTTTT TCTCCACACC | -1306 |
| CCCCACCCCC TGGTTTTCCC CACCCAGGGC CTAGGGCTGG AGGCCTGGGC | -1256 |
| CAGGGAGGTG GGGGAGGGAG AACGGGGCCT ACCGTGGTAT TAGATGTCTG | -1206 |
| AGTTTTGGTT GAGAGGGGAG CAAGGAACCT GATGTGCAGG TTCCATAGTG | -1156 |
| GAGGGGGCCC AAAGCGGGTG TCTTATCACT CTGTTTCAGC AAAGGTTGGG | -1106 |
| AAACTGAGGC CCAATCAGTC CAAAGTCTGG TCCCTTGAAG GGGAAGTAGG | -1056 |
| GGCCAACCCC TTAGTCTGTT AGATGAGGAG AGTCTGGAGT CTGATTCTGG | -1006 |
| AAGACGGAGG GGTGGGGGGA TGGGGGGGTG GGGGGATATA GCACGGAGGC | -956 |
| CTTGTCTGGC AGTCTACTCT TGAAGATGGG GTGAAATTTG GCAGGCTGGG | -906 |
| CAGATGGTGC CAGGCACCCA GGCTGCGGGG TGGCTGGATT TGGCCAGTAT | -856 |
| CGGGATGGGA ATGCCTAGGA TTCTGGATGG ATCGGGGGAA GGCATAAGGG | -806 |
| AGCAGCTGGC CATTGTGCTT ATGGCTGTTG ATGCATTGAG GGATAGCGCC | -756 |
| ACACACACAT TCAATAAATT TGAGGAGCTG AGAGGGTGAC TGGCCCCTGA | -706 |
| AGGCACAGTG CCAGAGGTCT GTGGAGAGGG GGTCAAGCAC CTGGGTTCCT | -656 |
| GAAGAACATG GAGGTGTGGG AGTGATTCCA GACAGCTGGG ATGTGCAGAG | -606 |
| CCTGAGAGAG TGCCAGGGAG CGGGTTGGGA GTTGAAAGTT GGGTGTGGTG | -556 |
| GCTCACGCCT TTAATCATGA CACTGGGCGG CAGAGGCGGG AGGATTTCTT | -506 |
| GAGGACAGGA ATTCAAGACC AGCCTGGGTA ACATAGCAAG GCCCCATCTC | -456 |
| TACTAAAAAT AAAAAAACTA ACAGGGCACA GTGGTCCAAG CCTGTAGTCC | -406 |
| CAGCCACTTA GGAGGCTGGA GCAGAAGGAT TGCTTTGGCC CAGTAGATCG | -356 |
| AGGCTACATT GAGCCATCAT TGTACTCCAC TGCACTCCAG TCTGGGCAAC | -306 |
| AAAGTGAGAC CCTGTCTTAA AAAATAAAAA TAAAAAAAGT TTCTGTGGGG | -256 |
| GACCTGCACT GAGGTCCTGG AGGGGCGCCA GTTGTGTCTC CCGGTTTTCC | -206 |
| CCTTCCACAG ACACCATTGC CACCACCATT AGGCAAACAT CCTTCGCCTC | -156 |
| AGTTTCTCCC CCCACCTCCC TCTCCTCCAC CCATCCAGGG GGCGGGGCCA | -106 |
| GAGGTCAAGG CTAGTGGGTG GGACTGGGGA GGGAGAGAGG GGTTGAGTAG | -56 |
| TCCCTTCGCA AGCCCTCATT TCACCAGGCC CCCGGCTTGG GGCGCCTTCC | -6 |
| TTCCCCATGG CGGGACACCT GGCTTCGGAT TTCGCCTTCT CGCCCCCTCC | 45 |
| AGGTGGTGGA GGTGATGGGC CAGGGGGGCC GGAGCCGGGC TGGGTTGATC | 95 |
| CTCGGACCTG GCTAAGCTTC CAAGGCCCTC CTGGAGGGCC AGGAATCGGG | 145 |
| CCGGGGGTTG GGCCAGGCTC TGAGGTGTGG GGGATTCCCC CATGCCCCCC | 195 |
| GCCGTATGAG TTCTGTGGGG GGATGGCGTA CTGTGGGCCC CAGGTTGGAG | 245 |
| TGGGGCTAGT GCCCCAAGGC GGCTTGGAGA CCTCTCAGCC TGAGGGCGAA | 295 |
| GCAGGAGTCG GGGTGGAGAG CAACTCCGAT GGGGCCTCCC CGGAGCCCTG | 345 |
| CACCGTCACC CCTGGTGCCG TGAAGCTGGA GAAGGAGAAG CTGGAGCAAA | 395 |
| ACCCGGAGGA GGCAAGTGAG CTTCGACGGG GTTGGGGTGT GGGGAGGTGG | 445 |
| TCATGACAGG GCAGCCTGAT GGGGAAGTGG TCACCTGCAG CTGCCCAGAC | 495 |
| CTGGCACCCA GGAGAGGAGC AGGCAGGGTC AGCTGCCCTG GCCAGGGAGG | 545 |
| GGTGTGTATC AACTGCAGGC AGCCCTGGCA GGCAGGGGCC AGGTGGGAAC | 595 |
| TGGAAGCTGG ATTTCGAAGA GACAACTGCC GGTGAGGGCA GAGCGGCCTG | 645 |
| GGAGAGTCGG AAGCTGGCCC AGGCTGGCCT TTGCTCTGTC TGGCCCAGCC | 695 |
| CTTGTCAGGG TCTCTCACAT CTCCTAGGCC TGCCCAGGGT CTGGTCACTC | 745 |
| ATTACTGGCC CAGCACCAGA CCCAGCTTGG GGTTGGTTTG AGCCCCTTTT | 795 |
| CCCACCCTTA GTCCTGCTTG AAAATTTGAC CCTTATCAGA CCCAAGATTT | 845 |
| TGGCCTTAGG GTTAAGCATA GCCTGAGGGT AAAAACAGTG CTCATTCCAG | 895 |
| GATTATTGTT CCTGAAAGTC TAGGGTGTGA CTCGTTTCTG ATAGGATCTC | 945 |
| CTGTTTGGGC TGTGTGTGTG CGCGTTGTGA GCTGGGTTTA CCTCCAGTCA | 995 |
| AGTATAGGGC TTGTCTTCCC CGGATCTCTG CCTCAGGCCA ATGACTGGCC | 1045 |
| ACTGTGTTAA GGTGCACACC CTGGCACCCC TTGTAGAAAG CTGGATTTTG | 1095 |
| ATTGACTTCA GCCTCAGTTC CAAAGTTGTA AACAAGAAAA ATGGTGAGAG | 1145 |
| ATTTCTCCAG GCCATTTGCA AATATAGAGC TGCTGCGGGA TTGAAGGCAT | 1195 |
| CCAGCCCTGC TGAGGACTAT TAAAGATGTA TCTTCCAGTC CTTCAAGGCG | 1245 |
| ACAAGTGTAA GCAATTAGAG ATTAAATACT AAGCCTTGAG ACCTCACAGA | 1295 |
| AAGGTGTGAC TGGTTTCTGG AGTGACCGAG AAGCCCCAAC CTCTTCGCAG | 1345 |
| GAGGTCACTG CTGAGCCTTG AATGATAATG GCTGGCAATT GTGGTCCACT | 1395 |
| TCCTAAGTGC CTGGCTGTGT GCTCCGTTTA TACATCATTA TCTCATTAAC | 1445 |
| CAGCACAAAA TCTCCTAGGG GGAGGTATTA TTATCCTATT TAACGGGTTT | 1495 |
| TAACTGCTAA ATGATGAAGC GAGGATTTGG ACCAGTGTTT ATTCCAAAAC | 1545 |
| CCCAAAACAG AATTTGGAAA ATCCAGGATA GCAGAGGGCA TTTATCAGTT | 1595 |
| TGAGTTATTG GCTGAGCAGA AGTTGGGGAT GAAAACAGCC TATTTGAAAT | 1645 |
| TGATATGATC AAGCACCATT GAAACACTTC CTTGAGGCTT CAGGACTACA | 1695 |
| AAAAGGCCTT GTTTTTTTCT CACTAGCTGT GCACCTCTGT CCGCCGGCAG | 1745 |
| CCTCATATGG CATGCCCCAG GGCTCAGTCC TTCAACCTCT GCTCTATCTA | 1795 |
| CCCTTCCTTC CTCTCACCCA CCCTCAAGGC TTAAATGCCA TTTAGACACC | 1845 |
| AGATGACTAC CGCGTTTTCT GTCTCTTGTG ATGGCTCCCT GAACTGCTCC | 1895 |
| ACCCTGATCA CCCAGTTGCT CAAGGCCAAA CCCAGTCATC CTCAGTTTCT | 1945 |
| TTCACGTCCT ACATCCTATC CTTAAGAAAC ATCCTGAATC AATCACAACC | 1995 |
| TAACCCTGGC CTCAGCCACC ATCATCTCTG CTGGGATTAC CGCAGTAGCT | 2045 |
| TCTCAAATTA TACTGCTTCC TCCCTACTGT CTGTGGCCAA CACGTCAACT | 2095 |
| AGAGTCAGTG TTTTAAAAGG TGTGGCCAGG CACTTTGGGA GGCCGAGGCA | 2145 |
| GGCGAATCAC CTGAGGTTGG GAGTTCGAGT CCAGCCTGAC CAACATGGCG | 2195 |
| AAACCCCATC TCTACTAAAA ATACAAAATT AGCTGGGCGT GGTGACGCAT | 2245 |
| GCCTGTAATC TCAGCTACTC AGGAAGCTGA GGCAGGAGAA TCGCTTGAAC | 2295 |
| CTGGGAGATG TAGGTTGCGG TGAGCCGAGA TCGCGCCAGT GCACTCCAGC | 2345 |
| CTAGGCAACA AAAGCGAAAC TCTCAAAAAA AAAANNAAAA AAGGTGAGGC | 2395 |
| TAGGTGCGGT GGTTCACACC TGTAATCCCA GCACTTTGGG AGGCCAAGGT | 2445 |
| GGACAGATCA CTTGAGGTCT CCTGACCAGC CTGGCCAACA TGGTGAAACC | 2495 |
| CCATATCTAC TAAAAATACA AAAATTAGCC GGGCATGGTG GTGGGTGCCT | 2545 |
| GTAGTCCCAG CTACTCAGGC GGCTGAGGCA GAATAGCTTG AACCCAGAAG | 2595 |
| GCGGAGATTG CAGTGAGCCA AGATCACGCC ACAGCACTCC AGCCTGGGCG | 2645 |
| ATAGAACGAG ATTCCGTCTT GGGGGGGGAG AAAAAGGGTG AGAGATCATT | 2695 |
| TCACTTGGAC TAAAACAAAG TCACTATGTC TGCAACAGGA TCTACCTAGC | 2745 |
| CACCAGACCA GCTTTGGGCT CTGGAAGGCC CACTTCAGGG CCTTGCCACA | 2795 |
| TTAGACTCTT GTCCTTTGCT CAAACAATCA CCTTCTCTGT CTTTAAAAGT | 2845 |
| GTCACCCTCC TCCATAATCT CCTTCCCTCC TTTACCCTAC TCCTATAGAC | 2895 |
| TGCTTTATTT TTTTTTTAAT TTTTGAGATG GAGTCTCACT CTGTCCCTCA | 2945 |
| GGCTGGAGTG CAGTGGTGCG ATCTTGGCTC ACTGCAAACT CCACCTCCTA | 2995 |
| GGTTCAAGCA ATTCTCCTGC CTCAGCCTCC TGAGTAGCTG GGATTATAGG | 3045 |
| GGAGCGCCAT GATGCCCAGC TAATTTTTGT ATTTTTAGTA GAGACAGAGT | 3095 |
| CTCACTATGT TGACCAGGCT AGTCTTGAAC TCCTGACCTC AAGTGATCTA | 3145 |
| CCCACCTTGG CCTCCCAAAG TGAAGGGATT ACAGGCATGA CCACTGCGCC | 3195 |
| CGGACTGCTT TACTTTTTTC CATAATATAT ATATATTTTT TAAATAGAGG | 3245 |
| CAGCAGGGGT GGGAGAAGGG GCGGCACGGG TCTCACTATG TTACCCAGGC | 3295 |
| TGCTTTCTAA CTCTTGGGCT CAAGCAGTCT GCCCACCTTG GCCTCCCAAA | 3345 |
| GTGCTAGGAT TTACAGACAT GAGCCACTGT GCCTGGCCAT TTTTTATTTT | 3395 |
| ATTTACTTTT TTATTTTTCA GAGCAGGAGT GGAAGTTTAT TATTAAAAAG | 3445 |
| TTATAGGGCA GGGAAAAAAG GAAAGTGCAC TTGGAAGAGA TCCAAGTGGG | 3495 |
| CAACTTGAAG AACAAGTGCC AAATAGCACT TCTGTCATGC TGGATGTCAG | 3545 |
| GGCTCTTTGT CCACTTTGTA TAGCCGCTGG CTTATAGAAG GTGCTCGATA | 3595 |
| AATCTCTTGA ATTTAAAAAT CAATTAGGAT GCCTCTATAG TGAAAAAGAT | 3645 |
| ACAGTAAAGA TGAGGGATAA TCAATTTAAA AAATGAGGAG TAAGTACACA | 3695 |
| CAAAGCACTT TATCCATTCT TATGACACCT GTTACTTTTT TGCTGTGTTT | 3745 |
| GTGTGTATGC ATGCCATGTT ATAGTTTGTG GGACCCTCAA AGCAAGCTGG | 3795 |
| GGAGAGTATA TACTGAATTT AGCTTCTGAG ACATGATGCT CTTCCTTTTT | 3845 |
| AATTAACCCA GAACTTAGCA GCTTATCTAT TTCTCTAATC TCAAAACATC | 3895 |
| CTTAAACTGG GGGTGATACT TGAGTGAGAG AATTTTGCAG GTATTAAATG | 3945 |
| AACTATCTTC TTTTTTTTTT TTCTTTGAGA CAGAGTCTTG CTCTGTCACC | 3995 |
| CAGGCTGGAG TGCAGTGGCG TGATCTCAGC TCACTGCAAC CTCCGCCTCC | 4045 |
| CGGGTTCAAG TGATTCTCCT GCCTCAGCCT CCTGAGTAGC TGGGATTACA | 4095 |
| GGTGCGTGCC ACCGTGCCCA GCTAATTTTT GTGTTTTTAG TAGAGACGGG | 4145 |
| GTTTCACCAT GTTGGCCATG CTGGTCTTGA ACTCCTGACC TCGTGATCTG | 4195 |
| CCCACCTCGG CCTCCCAAAG TGCTGGAATT ATAGGCGTGA GCCACCGCGC | 4245 |
| CCAGCAAAGA ACTTCTAACC TTCATAACCT GACAGGTGTT CTCCTCGAGG | 4295 |
| CCAGGGTCTC TCTTTCTGTC CTTTCACGAT GCTCTGCATC CCTTGGATGT | 4345 |
| GCCAGTTTCT GGGGGAAGAG TAGTCCTTTG TTACATGCAT GAGTCAGTGA | 4395 |
| ACAGGGAATG GGTGAATGAC ATTTGTGGGT AGGTTATTTC TAGAAGTTAG | 4445 |
| GTGGGCAGCT TGGAAGGCAG ATGCACTTCT ACAGACTATT CCTTGGGGCC | 4495 |
| ACACGTAGGT TCTTGAATCC CGAATGGAAA GGGGAGATTG ATAACTGGTG | 4545 |
| TGTTTATGTT CTTACAAGTC TTCTGCCTTT TAAAATCCAG TCCCAGGACA | 4595 |
| TCAAAGCTCT GCAGAAAGAA CTCGAGCAAT TTGCCAAGCT CCTGAAGCAG | 4645 |
| AAGAGGATCA CCCTGGGATA TACACAGGCC GATGTGGGGC TCACCCTGGG | 4695 |
| GGTTCTATTT GGTGGGTTCC CCTCTGCAGA TTCTGACCGC ATCTCCCCTC | 4745 |
| TAAGGAGTAT CCCTGAACCT AGTGGGGAGG GGCAGGGGCA GACTCTACCC | 4795 |
| TCACCCATGA AGAGGAGTAG GGAGAGGGAG AAGATGCTTG GGCTTTGAGC | 4845 |
| TCCCTCTGGG AAGAGGTGGT AAGCTTGGAT CTCAGGGTCA CAAGGGCCCT | 4895 |
| GCGTGCTCCC TCATTTTGCT TCTCTTTTGA CTGGCCTCCC CCAGGGAAGG | 4945 |
| TATTCAGCCA AACGACCATC TGCCGCTTTG AGGCTCTGCA GCTTAGCTTC | 4995 |
| AAGAACATGT GTAAGCTGCG GCCCTTGCTG CAGAAGTGGG TGGAGGAAGC | 5045 |
| TGACAACAAT GAAAATCTTC AGGAGGTAAG GGTGGGAGGG GGATACCCGG | 5095 |
| GGACCTTCCC TTTCTTGGCC TAATTTCCAT TGCTTCCATC ACTGGCTCGT | 5145 |
| AGCTCTCCGT CTTTGGTGCA GTGGTTCTCA GTGGGATGGA GTGAAATTCC | 5195 |
| TCAGTTCTGC TGGGATAAGG TCCAGAGCCA ACCCTTCCAG GATCCTGCCT | 5245 |
| TTTCACACCA CCACCTGGCT CTGCTGACAC ATCTAGTCAC AGACCCCTGT | 5295 |
| GATGCTGTTA CTCAGCAAGT CCAAAGCTTG CCCTTGTCAC CCCCTTCCCA | 5345 |
| CCTGCACAGA TATGCAAAGC AGAAACCCTC GTGCAGGCCC GAAAGAGAAA | 5395 |
| GCGAACCAGT ATCGAGAACC GAGTGAGAGG CAACCTGGAG AATTTGTTCC | 5445 |
| TGCAGTGCCC GAAACCCACA CTGCAGCAGA TCAGCCACAT CGCCCAGCAG | 5495 |
| CTTGGGCTCG AGAAGGATGT GAGTGCCATG TCTCTCTGCG GGCTCCATCT | 5545 |
| CTTTCCCCTG TCACCACCTC GCTTTCCCTA GCTCTGGCTC CTCCAACTGC | 5595 |
| TCTAGGGCTG TTGGCTTTGG ACAGAATGTC CAAGCAGTCA GGCCTGTCTC | 5645 |
| AGCTCATTCT CTAATGTCCT CCTCTAACTG CTCTAGGGCT GTTGGCTTTG | 5695 |
| GATAGAATGT CCAAGCAGAG TCAGGCCCGT CTCTCAGCTC ATTGTCTAAT | 5745 |
| GTCATTCTCC TTTCTGTCAT TCACTGGCAG GTGGTCCGAG TGTGGTTCTG | 5795 |
| TAACCGGCGC CAGAAGGGCA AGCGATCAAG CAGCGACTAT GCACAACGAG | 5845 |
| AGGATTTTGA GGCTGCTGGG TCTCCTTTCT CAGGGGGACC AGTGTCCTTT | 5895 |
| CCTCTGGCCC CAGGGCCCCA TTTTGGTACC CCAGGCTATG GGAGCCCTCA | 5945 |
| CTTCACTGCA CTGTACTCCT CGGTCCCTTT CCCTGAGGGG GAAGCCTTTC | 5995 |
| CCCCTGTCTC TGTCACCACT CTGGGCTCTC CCATGCATTC AAACTGAGGT | 6045 |
| GCCTGCCCTT CTAGGAATGG GGGACAGGGG GAGGGGAGGA GCTAGGGAAA | 6095 |
| GAAAACCTGG AGTTTGTGCC AGGGTTTTTG GGATTAAGTT CTTCATTCAC | 6145 |
| TAAGGAAGGA ATTGGGAACA CAAAGGGTGG GGGCAGGGGA GTTTGGGGCA | 6195 |
| ACTGGTTGGA GGGAAGGTGA AGTTCAATGA TGCTCTTGAT TTTAATCCCA | 6245 |
| CATCATGTAT CACTTTTTTC TTAAATAAAG AAGCTTGGGA CACAGTAGAT | 6295 |
| AGACACACTT ATCTTGGTTT GTCCTTCAGT TACTGAGGTG GGGATGGGAA | 6345 |
| TATCCAATGC TCATACCCAA GTGACCCTGA AACTAAGGTG CCATTTACAC | 6395 |
| TCCTTAAGGT CACACAACAT CAGAGGGAGA GCTGGGATTG CAGCCAAGTT | 6445 |
| TATTTGTACA GGGCCCTGTG ATAGGCTAGT TCCCAAAAGC CTGTGATGCA | 6495 |
| AGAACTTTTG CCCATAGACT CAGTCACCAT GTAGCTGTTA CCTGTTCAGA | 6545 |
| GCTGGCTTTT TGCTTTCCCA CCCTACTCTG GAATTCTTAA ATGGCTTTAT | 6595 |
| ACTTAGAAAT CATCTTATTT CTGTTGAACC TAGATCACCC CAACCAGAAA | 6645 |
| CTTCTATTAA TACTTTGTGC TTTCTTGATA CCAGGGTCTA TTTGGTTTCC | 6695 |
| ACTTAAGGTT TTTGCATACT CTGCCCATAA GTGACTCATT AGTTACTCAA | 6745 |
| GTTTTATTCC TGGCTCTGCC ACTAGTTCAT TAGGGGTCTT TGCCCCAGAG | 6795 |
| TCATTTCTTC CATGTAAAAA AACTTGGGCT CATTAAATCT AGGTAGGAAA | 6845 |
| GGGCGGATGT GGCAGGTTTT AATAGAACAG GTCAAGATAA GGCTTTATTT | 6895 |
| CTATAGAAAT GATGCTTTGA CAATAGTTTG GCTTGGTGTA AGGCTCACAA | 6945 |
| AAGAAAATCA CATGTACCAT GTGTGGGTTA AGCGGTTTGA TTCACACTGA | 6995 |
| ACCAGGCCAG CCCAGTTGCC CTCTGCTGTG TCCACCCGTG GAGTGGAGCT | 7045 |
| GTGTCACAGC CATCACACTG GTAAACTGCT GTAGCTGGTT TACCAGGCTT | 7095 |
| TCTCTTGCCC TGACAGTACA GGTGAAGCCT GTAAATAAAT CTTCTGCTAT | 7145 |
| CTTTGTGAAC TTAACCAAAT CCCAGTTACC TTATTTAAAT GGCAATAGAT | 7195 |
| CTGTTTTCCC TTAAACTAGA AACCTTAATT ACCTGTATTC CTACCTCCAG | 7245 |
| CTCAACCCAT ATATTTGCAC CTTTCCAGTA AGCAGGTTTG TATTTCCATC | 7295 |
| TCTCCCCTTC CCCTAAGATT CTGAATTAGT TCTCCAGACC TTGCCAAGCA | 7345 |
| CATTCTCCCT GGAAAGCAAG GAATATCACA GACCCACAAG AAGAGTAAAT | 7395 |
| GCCCAGGAGT GAATGAAGCG GCTTGTCCTT GACTTGGAAA TAAAAGCAAA | 7445 |
| GCTGTGAAAA GCCAGGTCGC TACGATTTTG TTGGCAGCAG GACTAGCCAC | 7495 |
| AGAGTAGGGA AGTTTTGGGG CCAGGCCCTA GGTTTTCCCA GAATGCCTTG | 7545 |
| GGTGATGCCA CCAAGAACCT TAAGAACTCC CTCTTACATT TTCCATCGGT | 7595 |
| TGCAGGCATG GCTTTTGCAA CTGATAGGTG CTCTGACTAC AGATATTCTG | 7645 |
| GTGTCCATGG CAACATGGCC TTATGGCTTG TAATAGTGGG AACTTCCAGC | 7695 |
| TCACTGGCAA TTTCCTGGAG GTGGCAATAT CTTTGGCAGG AAACCATCTC | 7745 |
| TTCTGTCTTG GCCACCAGGG TCTGCTCCAG GCCCTGAATG AGTCCACATC | 7795 |
| AATCCATCCT TCTTCCCTGT AGTCATTCCC TCCTGGGGAG GGAAGGGAAG | 7845 |
| GGGGGTATTT ATCAGACCTA TTTGCTCGCT GCTGTGTCTA CTCATGGGCA | 7895 |
| GGTGTGTCCG ATGGTGCCTT GGCGGTGGAC CTTAGGTCTG AATGCCAGCC | 7945 |
| CGGCACCCTG GGCATCGGAA GTCGGCCTCC CTGGCAGCCT GGATGCTGCA | 7995 |
| GCCAACACAG GCCACATGGA ACCAGACGTC ACAGCCATCA CACTGAACCC | 8045 |
| AGGCCACTGT CTCTTCCTGG GGCAGGCAGC AACAAGGAGC TGCACAGGGC | 8095 |
| TCCCCGCCCC CAACTGCAGG GGGAGCCATG GGAGCGCTCA CTGGGTACTT | 8145 |
| CCGAGGACGG CCACGTCGAT TTCTGAAGGA AGTGACACAG ACACAAGGGT | 8195 |
| CACCGGAAAC CTGTAGGAGA AGGCCTAGAC CTTGCCACCC TATGGCTGGT | 8245 |
| CATAGGGTGA GGCCACAGAG GCACAGTGGT CTGAGAACTA AGGTGAGCCT | 8295 |
| GGACTCTGTC C |  |
